# The Maize OST1 Kinase Homolog Phosphorylates and Regulates the Maize SNAC1-Type Transcription Factor

**DOI:** 10.1371/journal.pone.0058105

**Published:** 2013-02-28

**Authors:** Belmiro Vilela, Alicia Moreno-Cortés, Agnese Rabissi, Jeffrey Leung, Montserrat Pagès, Victoria Lumbreras

**Affiliations:** 1 Centre for Research in Agricultural Genomics, Bellaterra, Cerdanyola del Vallés, Spain; 2 Institut de Sciences du Végétal, Centre national de la recherche scientifique, Gif-sur-Yvette, France; National Taiwan University, Taiwan

## Abstract

The *Arabidopsis* kinase OPEN STOMATA 1 (OST1) plays a key role in regulating drought stress signalling, particularly stomatal closure. We have identified and investigated the functions of the OST1 ortholog in *Z. mays* (ZmOST1). Ectopic expression of *ZmOST1* in the *Arabidopsis ost1* mutant restores the stomatal closure phenotype in response to drought. Furthermore, we have identified the transcription factor, ZmSNAC1, which is directly phosphorylated by ZmOST1 with implications on its localization and protein stability. Interestingly, ZmSNAC1 binds to the ABA-box of ZmOST1, which is conserved in SnRK2s activated by ABA and is part of the contact site for the negative-regulating clade A PP2C phosphatases. Taken together, our results indicate that ZmSNAC1 is a substrate of ZmOST1 and delineate a novel osmotic stress transcriptional pathway in maize.

## Introduction

Plant growth and crop productivity are compromised by environmental stresses such as extreme temperatures, drought and high salinity. To cope with these adverse situations, plants have developed complex signalling networks to regulate physiological and biochemical acclimation. Reversible protein phosphorylation is one of the major mechanisms for modulating intracellular adaptations, in particular, those involved in ABA and stress signalling [1]–[3]. The ABA signal can stimulate – within minutes – regulators such as Sucrose non-fermenting related kinase (SnRK)-2 subfamily [4] that are central to diverse physiological responses.

Members of the SnRK2 subfamily have been characterized in different plant species [4]–[7]. The first SnRK2 gene, *PKABA* from wheat, is up-regulated by drought and ABA in seeds and vegetative tissues [7]. The homolog AAPK from faba bean is essential in relaying the ABA signal in stomatal closure [5]. Ten SnRK2 genes exist in the *Arabidopsis* and rice genomes. In *Arabidopsis*, except SnRK2.9, the kinase activity of each member of the family is activated by hyperosmotic stress [8] with five, SnRK2.2, SnRK2.3, SnRK2.6/OST1/SRK2E, SnRK2.7 and SnRK2.8 also activated by ABA [8]. A similar situation occurs in rice, in that the activities of three of the ten SnRK2 homologs (called SAPKs) are also stimulated by ABA [9]. Studies of mutants deficient in SnRK2.2 and SnRK2.3 activities showed that these kinases are required for ABA-mediated seed germination, dormancy and seedling growth but have minor roles in stomatal control [10]. Conversely, SnRK2.6/OST1, the ortholog of AAPK, is mainly involved in ABA-mediated stomatal closure in response to humidity decrease but only has a minor role during seed germination [4], [6], [11], [12]. In maize, eleven *SnRK2* genes have been reported [13], although only functional data are available for one, the corresponding protein ZmSAPK8/ZmOST1. This kinase, with a primary sequence highly homologous to that of the *Arabidopsis* OST1, is required in diverse stress signal transduction pathways, principally in drought and salt tolerance responses [13], [14].

The molecular mechanisms of OST1 in orchestrating both the “fast” (ion transport across membrane) and “slow” (gene expression) ABA responses are increasingly being understood, which has been particularly aided by the identification of direct targets. OST1 (i) activates the slow anion channel SLAC1 to trigger plasma membrane depolarization necessary to initiate stomatal closing [15]–[17], (ii) possibly inhibits the inward-rectifying K^+^ channel KAT1 [18], (iii) induces the generation of reactive oxygen species (ROS) that leads to Ca^2+^ spikes *via* the respiratory burst oxidase homolog F (RbohF) [19] and (iv) activates the bZIP-class of transcription factors that include ABI5 [20], [21] and the ABA-responsive element binding factors, ABF2 and ABF3 [11], [22]. ABFs 2, 3 and 4 together are thought to regulate about 80% of the global ABA dependent transcriptome [20], [22]–[24].

In comparison to ion transport across membranes [25]–[27], much less is known about the mechanisms of transcriptional control by ABA in guard cell signalling [28], [29]. Despite the apparent importance of the three ABFs as global regulators of the ABA transcriptome, the *Arabidopsis abf2/abf3/abf4* triple mutant is normal in transpiration [23]. This suggests that the ABF class of transcription factors is not critical for stomatal functions and that OST1 may have additional transcriptional targets [30]–[33].

In searching for cognate targets of the maize OST1 ortholog, we focused on the ABA/drought-inducible members of the NAC domain-containing transcription factors (SNAC proteins). Note that SNACs seem to have significant importance in stomatal adaptive regulation and also implications for improving plant stress tolerance [31], [34]. Overexpression of *SNAC* genes in different plant species ranging from *Arabidopsis* to maize leads to enhanced drought and salt tolerance [12], [35]–[41]. Of particular interest is the rice SNAC1, which is highly induced in guard cells by drought [35]. Similar overexpression of this protein in rice enhances plant drought tolerance under field conditions at the reproductive stage. It also improves both drought and salt tolerance at the vegetative stage, and more importantly, without yield penalty [35] and ZmSNAC1 has been described as a stress-responsive factor acting in positive modulation of abiotic stress tolerance [42]. Thus, SNAC factors are emerging as important nodes in osmotic stress signalling and as promising tools to engineer enhanced tolerance responses in plants with little compromise in biomass yield.

Maize (*Zea mays*) is an important food and feed crop worldwide, with more than 800 million tons cultivated annually, and about 130 million tons in the USA also being diverted for energy. However, maize requires high water input. For this reason, characterization of osmotic stress signalling pathways and proteins involved in maize water homeostasis are of huge economic importance as bouts of water shortage are becoming more frequent. In the present work, we have characterized the maize ortholog of the *Arabidopsis* OST1. We have also identified its cognate substrate, a SNAC-type transcription factor, ZmSNAC1. ZmSNAC1 represents a founding member of a new class of target that opens the possibility to better understand how ABA mediates transcriptional control of stomatal closure.

## Results

### ZmOST1 kinase can substitute the *Arabidopsis* OST1 in guard cell drought stress signalling

**Figure 1:**
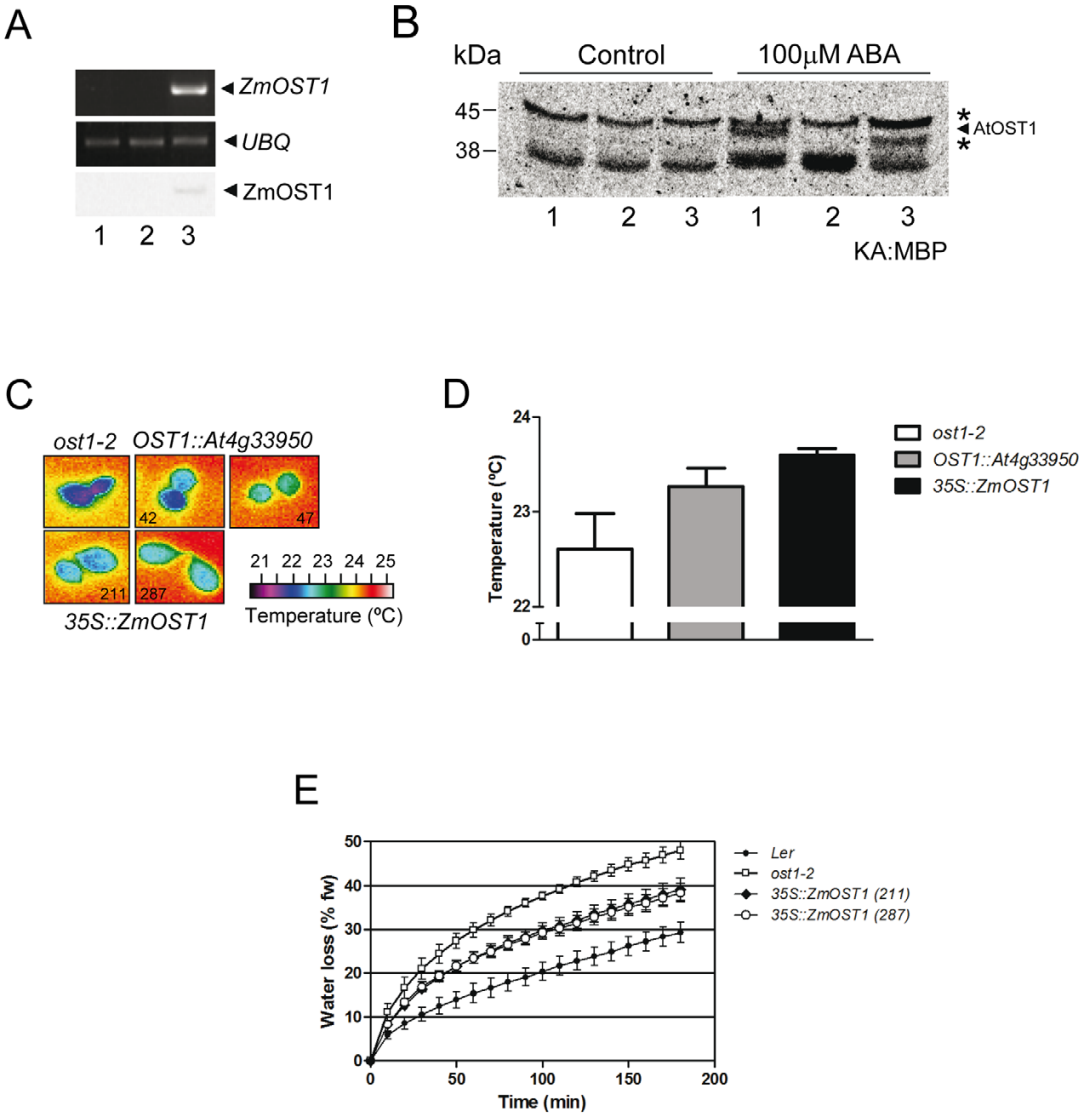
ZmOST1 complements the lack of OST1 function in drought stress signalling. (**A**) *ZmOST1* expression in different *Arabidopsis* lines analyzed by RT-PCR using *Ubiquitin* expression as control and by western-blot using an anti-ZmOST1 antibody. Lane 1, Ler wild-type seedlings; lane 2, *ost1-2* mutant; lane 3, *35S::ZmOST1/ost1-2* transgenic line (211). (**B**) OST1 activation by ABA in *Arabidopsis* protein extract analyzed by MBP in-gel kinase assay (KA: MBP). Lane 1, Ler wild-type seedlings; lane 2, *ost1-2* mutant; lane 3, *35S::ZmOST1/ost1-2* transgenic line (211). Sizes of molecular markers are shown on the left. Black arrow marks AtOST1 activity. Asterisks mark two new bands of ABA-induced kinase activities resulting from *ZmOST1* transgene expression. (**C**) Phenotypic *ost1-2* complementation by ZmOST1 in drought signalling. Detached leaves from *ost1-2* allele, *OST1::At4g33950/ost1-2* and *35S::ZmOST1/ost1-2* transgenic lines were monitored for foliar temperature by false-colour infrared image subjected to mild drought treatment [6]. (**D**) Quantification of infrared images. The average leaf temperatures were 22.61±0.37 for the *ost1-2* allele; 23.27±0.19 for *OST1::At4g33950/ost1-2* and 23.60±0.07 for *35S::ZmOST1/ost1-2*. (**E**) Water loss kinetics using detached leaves of wild-type (closed circles), *ost1-2* mutant (open squares) and two independent *35S::ZmOST1/ost1-2* transgenic lines (line 211, closed diamonds and line 287, open circles). Water loss is expressed as the percentage of initial fresh weight. Values are means ± SD of three independent experiments.

OST1 in *Arabidopsis* limits water loss in leaves through the regulation of stomatal closure [4], [6]. Since ZmOST1 shares a 83% sequence identity and a similar constitutive pattern of expression during development and stress responses with its *Arabidopsis* counterpart (Figures S1 and S2) we tested whether ZmOST1 is a functional OST1 ortholog by examining its ability to complement the severe *ost1-2* allele carrying the point mutation (G33R) in the ATP-binding loop domain [6]. The expression levels of the *ZmOST1* transgene and protein in *ost1-2* transgenic plants were analyzed by RT-PCR and western-blot analyses using ZmOST1 antibody (Figure 1A). The ZmOST1 activity in the complemented *Arabidopsis* plants was determined by MBP in-gel kinase assays (Figure 1B). It has been reported that ABA activates OST1 but that this kinase activity is missing in the *ost1-2* allele [6]. Comparing MBP phosphorylation from wild-type, *ost1-2* and *ZmOST1*/*ost1-2* seedlings we detected in the *ZmOST1*/*ost1-2* extracts a new specific ABA-dependent kinase activity that is absent in the mutant [6]. The 42–43 kDa activity is coincidental to the expected size of ZmOST1, suggesting that the maize kinase is active in *Arabidopsis* (Figure 1B).

The ability of ZmOST1 to functionally substitute the *ost1-2* mutation was assessed by comparing the temperature of detached leaves from *ost1-2* and *ZmOST1*/*ost1-2* plants by infrared thermography (Figure 1C and 1D). The *ost1-2* mutant transpires excessively due to its inability to close stomates triggered by drought, which leads to cooling of the leaf temperature [6]. We detected a temperature of 22.6°C in detached leaves from the mutant. By contrast, leaf temperatures of *35S::ZmOST1/ost1-2* plants were ∼0.5 to 1.0°C warmer (23.6°C), indicating the ability of *ZmOST1* to partially limit transpiration in response to drought. Surprisingly, the *ost1-2* mutant expressing the wild-type *Arabidopsis OST1* transgene under the control of its own promoter (*pOST1::At4g33950*) also only rescued partially the leaf temperature phenotype [6]. To confirm these results, water loss kinetics were performed on detached rosettes. Indeed, as compared to *ost1-*2, the water loss susceptibility in *35S::ZmOST1/ost1-2* rosettes was reduced again to 47–53% confirming the rescue of the *ost1-2* phenotype (Figure 1E). The partial complementation obtained in both experiments suggests that the endogenous mutated OST1-2 protein interferes with OST1 and ZmOST1 in the rescue of the ABA signalling pathway. An analogous phenomenon was observed with the inactive AAPK-kinase expressed in wild-type *Vicia faba* guard cells, which interfered with ABA responsiveness [4], [5], [11]. Taken together these results confirm that ZmOST1 is functionally conserved across monocots and dicots and support the hypothesis that ZmOST1 is a positive regulator of water deficit signalling in guard cells.

### ZmOST1 interacts with a homolog of the rice transcription factor SNAC1 *in vitro*

**Figure 2:**
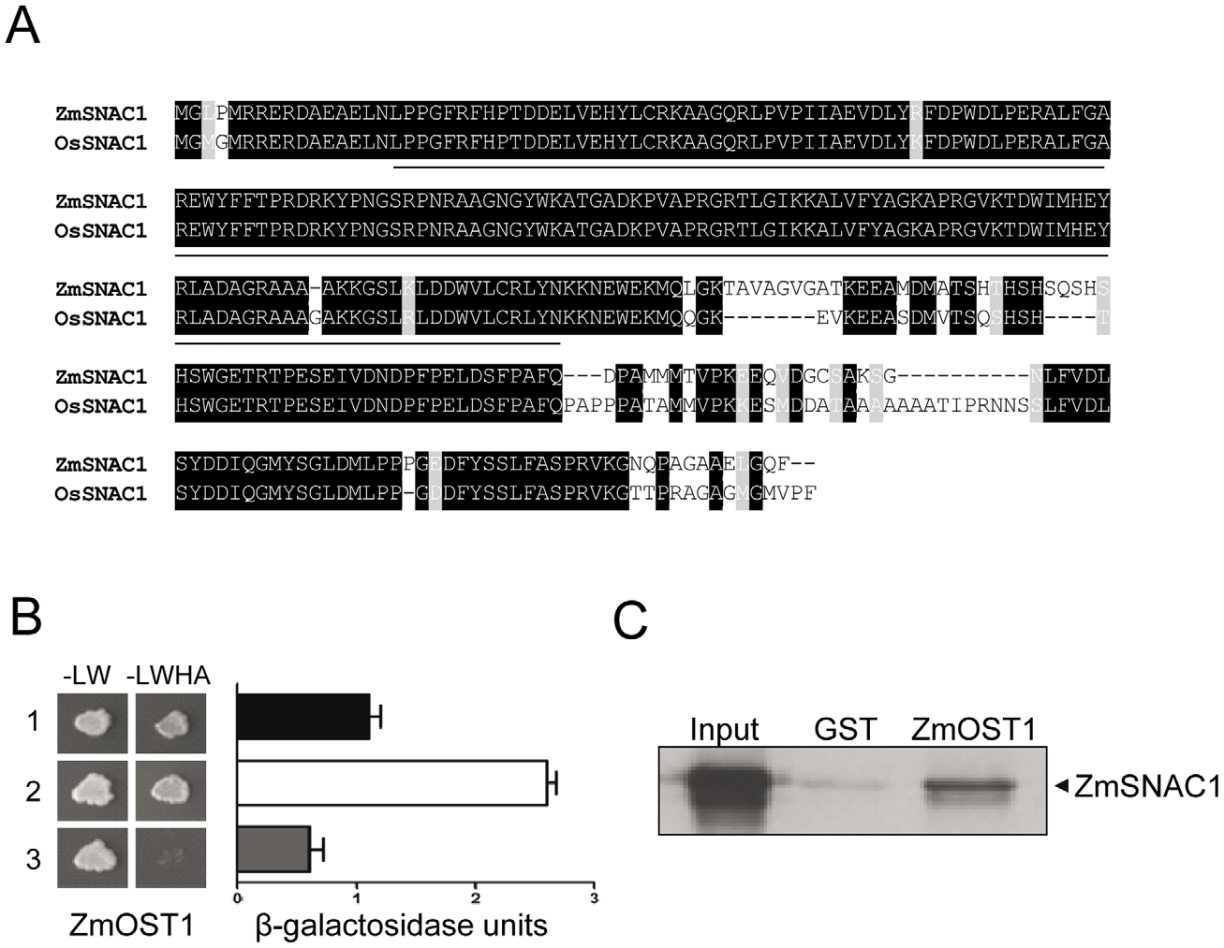
ZmOST1 interacts with ZmSNAC1. (**A**) Sequence alignment of maize and rice ZmSNAC1 proteins. The NAC-binding DNA domain is underlined below the alignment. (**B**) ZmOST1/ZmSNAC1 yeast two-hybrid interaction by growth in selective medium (left). β-galactosidase activity quantification of the co-transformed yeasts (right). Values are means ± SD of three independent experiments. 1, pGBT7-ZmOST1/pGAD424-ZmSNAC1 interaction; 2, α and β CK2 subunits interaction used as positive control; 3, pGBT7-ZmOST1/pGAD424 interaction as a negative control. (**C**) ZmOST1/ZmSNAC1 interaction is confirmed by *in vitro* pull-down assay. Equal amounts of labelled ZmSNAC1 were incubated with GST and GST-ZmOST1 proteins coupled to gluthathione-sepharose resin obtaining ZmSNAC1 specific *in vitro* retention.

To identify unknown ZmOST1 targets, we performed a yeast two-hybrid screen using ZmOST1 as the bait, and as prey, a cDNA library from maize leaves dehydrated for 3 hours. Among several positives clones, we focused on a SNAC1-related transcription factor for further characterization. The *ZmSNAC1* clone encodes a protein of 312 amino acids highly homologous to rice SNAC1 (Figure 2A) which functions mainly in stomatal regulation [35]. Both proteins are almost identical in their DNA-binding NAC domains suggesting that they are functionally conserved.

We then validated the interaction in yeast and found that co-expression of pGBT7-ZmOST1 and pGAD424-ZmSNAC1 proteins permitted yeast growth on selective medium and specific activation of the LacZ reporter system (Figure 2B). In addition, Figure 2C shows that the bacterially-produced GST-ZmOST1 fusion protein interacts with ZmSNAC1 transcribed *in vitro* but not with GST alone indicating that ZmSNAC1 is a direct target of ZmOST1.

### ZmOST1 interacts with ZmSNAC1 *in vivo*

**Figure 3:**
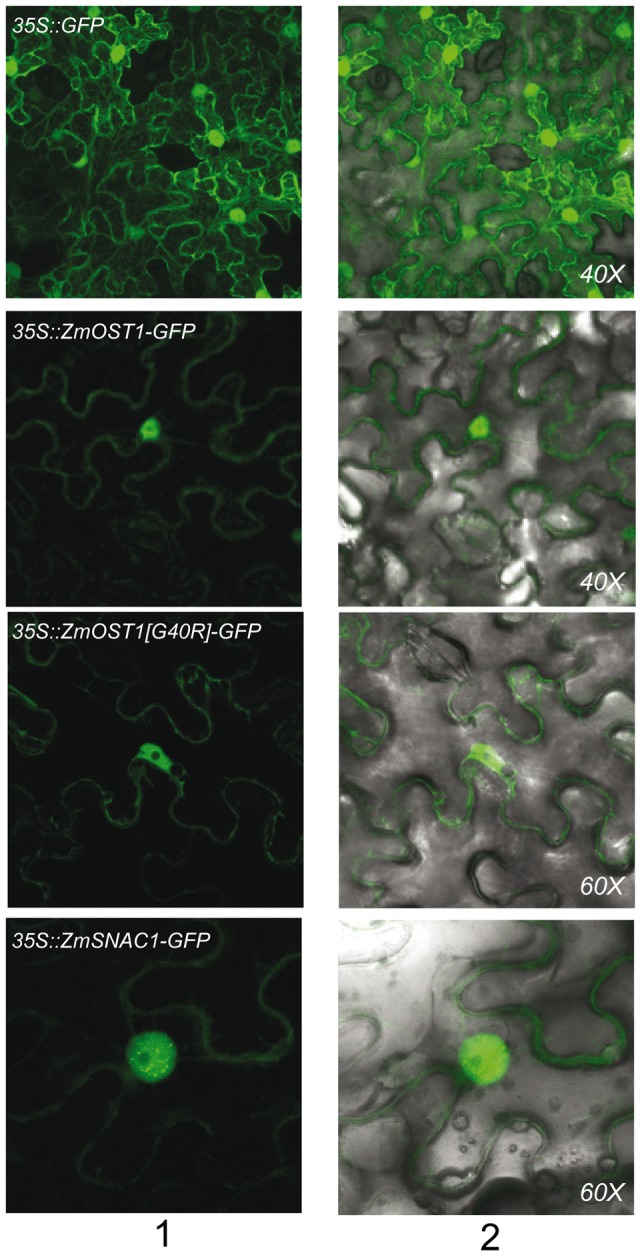
ZmOST1 and ZmSNAC1 co-localize in the nucleus. GFP, ZmOST1-GFP, ZmOST1 [G40R]-GFP and ZmSNAC1-GFP fusion proteins were localized by transient expression in epidermal tobacco leaves. Left, GFP signal; right, light microscope/GFP channel overlay.

We monitored the subcellular localization of ZmOST1-GFP and ZmSNAC1-GFP constructs in *Nicotiana benthamiana* and found that both proteins are localized in the nucleus and the cytoplasm of tobacco epidermal cells (Figure 3). Clade A PP2C phosphatases are upstream negative regulators of OST1 that constitutively inhibit its activity in the absence of ABA. In *Arabidopsis*, the mutated PP2C, *abi1-1*, has been shown to require nuclear localization to block stomatal closure [43]. We thus tested whether the subcellular localization of an inactive ZmOST1 kinase (Figure S3) with the point mutation G40R might be altered. This mutated form, however, maintains the same subcellular localization of the wild-type kinase. Unexpectedly, while the level of the wild-type ZmOST1-GFP is low, this mutated form accumulates to higher levels in transformed tobacco cells. Since there is no noticeable altered subcellular localization between the wild-type and the mutant ZmOST1, which is stable, this could explain why the kinases OST1-2 and AAPK similarly mutated in the P-loop could prevent full phenotypic complementation by their respective wild-type counter parts.

**Figure 4:**
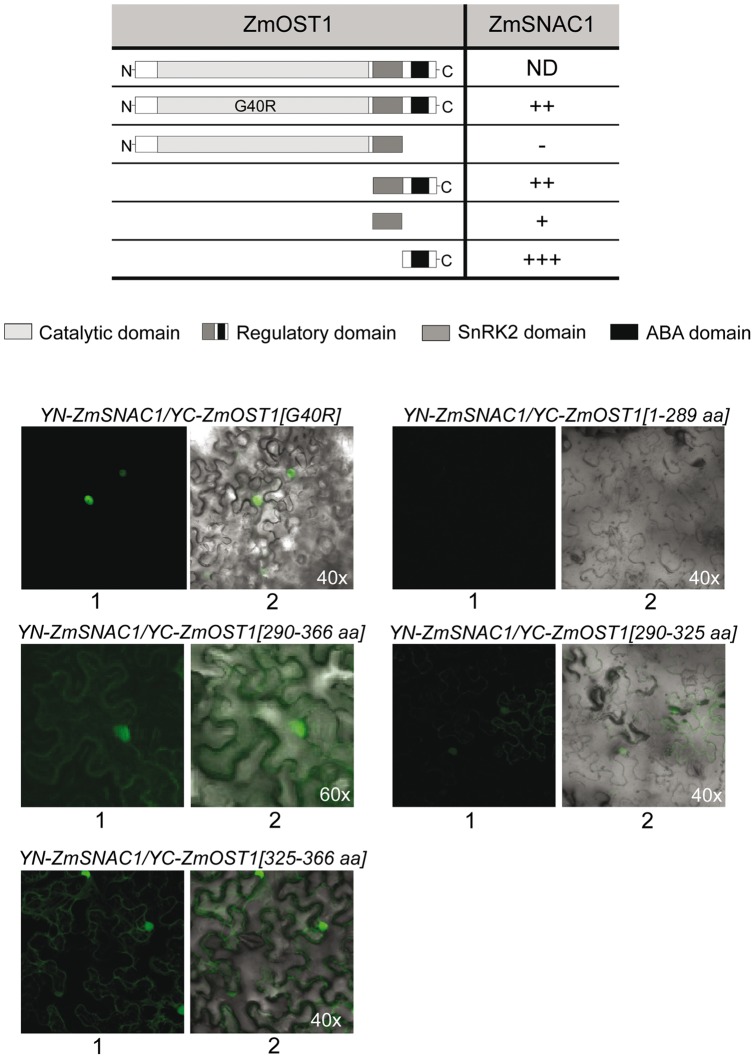
The interaction of ZmOST1 with SNAC1 depends on the ZmOST1 ABA-box. BiFC analysis of the interaction between ZmSNAC1 and different ZmOST1, ZmOST1 [G40R] mutant, and deletion forms as depicted on the left. Relative quantification of the BiFC interaction is shown on the right. BiFC fluorescence images analyzed by confocal microscopy are presented on the bottom. 1, YFP signals; 2, light microscope/BiFC channel. Numbers indicate ZmOST1 amino acid regions included in each construct.

Next, we used bimolecular fluorescence complementation (BiFC) [35], [44]–[46] to determine whether and where ZmSNAC1 interacts with ZmOST1 *in planta*, and if so, to characterize the specific ZmOST1 domains involved in this interaction. Full-length ZmOST1 (G40R) and different deleted derivatives were fused to the C-terminal half of the YFP while the ZmSNAC1 factor was fused to the N-terminal half (Figure 4). The results showed that ZmOST1 interaction with ZmSNAC1 is mainly nuclear and the different derivatives showed a nuclear/cytosolic distribution (Figure 4). ZmOST1 interaction is independent of the kinase activity and is mediated by a site in the C-terminal regulatory domain corresponding to the region between amino acids 290–366. This domain is present in ABA-dependent SnRK2 kinases and is important for the negative regulation by the clade A PP2C phosphatases [47]–[49]. As shown in Figure 4, co-expression of the regulatory domain or the ABA-box of ZmOST1 alone, amino acids 325–366, with ZmSNAC1 reconstituted the YFP signal. No interaction was detected between *YN-ZmSNAC1* and *YC-ZmOST1 (1*–*289aa)* constructs. Thus, the ABA box is necessary and sufficient for this interaction [49]. While the ABA-box has been shown to form part of the contact site for the negative regulating PP2Cs [49], [50], our results reveal that it is also important for substrate-binding. This raises the possibility that ZmSNAC1 may compete with the clade A PP2C phosphatases sharing the same docking region, highlighting the interesting perspective of substrate occupancy as a mechanism to sustain ABA signalling [48].

### ZmSNAC1 is phosphorylated by ZmOST1

**Figure 5:**
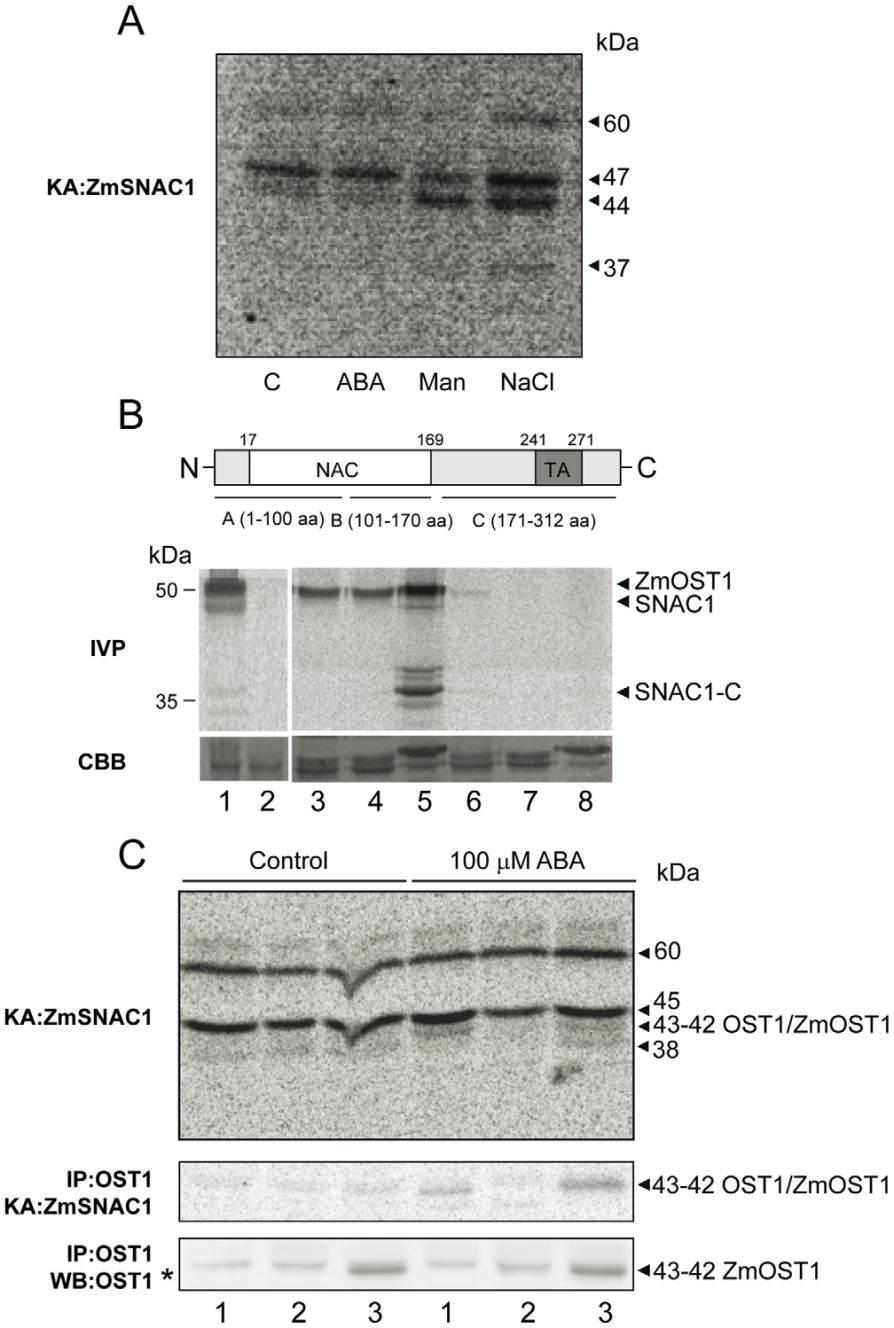
ZmSNAC1 phosphorylation by osmotic stress is dependent on ZmOST1 activity. (**A**) ZmSNAC1 phosphorylation is analyzed by in gel kinase assay (KA: ZmSNAC1). Protein extracts were prepared from maize young seedlings 30 min after treatments with MS 0.5× medium (C), 100 µM ABA (ABA), 400 mM mannitol (Man) and 250 mM salt (NaCl). Sizes of the activity kinase bands obtained are shown on the right. (**B**) ZmSNAC1 *in vitro* phosphorylation by ZmOST1 (IVP). Schematic representation of ZmSNAC1 domains used in the experiment. Numbers indicate ZmSNAC1 amino acid regions included in each construct. AD marks the activation domain [35]. Phosphorylation of ZmSNAC1 and deletion forms is tested *in vitro*. 1, ZmSNAC1 phosphorylation by ZmOST1; 2, ZmSNAC1 alone; 3, 4, 5; ZmOST1 phosphorylation of ZmSNAC1 fragments A, B and C, respectively; 6, 7, 8 ZmSNAC1 fragments A, B and C, respectively. The expression of the different ZmSNAC1 constructs is verified by Coomassie-blue protein staining (CBB). (**C**) In gel kinase assay with proteins extracted from 7 day-old seedlings treated or not with 100 µM ABA (KA: ZmSNAC1; upper gel). In gel kinase assay after immunoprecipitation of the same samples with an antibody against the ZmOST1 ABA-domain (IP: OST1/KA: ZmSNAC1; centre gel). Western-blot of the immunoprecipitation experiment (IP: OST1/WB: OST1; lower gel). Lane 1, Ler wild-type seedlings; lane 2, *ost1-2* mutant; lane 3, *35S::ZmOST1/ost1-2* transgenic line. The ZmSNAC1 protein was used as substrate. Sizes of the activity kinase bands obtained are shown on the right.

If ZmOST1 is activated by ABA and hyperosmotic stress, we reasoned that these treatments may lead to ZmSNAC1 phosphorylation. In fact, even though the optimal motif for OST1 phosphorylation, LXRXX(S/T) [48], is absent in the ZmSNAC1 primary sequence, we were able to predict potential phosphorylation sites using a web-based bioinformatics tool (Table S1) [51]. To test ZmSNAC1 phosphorylation we used maize extracts pre-treated or not with either ABA, mannitol or salt to detect kinase activities toward recombinant ZmSNAC1 protein. Using in-gel kinase assays our results revealed a 43–44 kDa kinase that was rapidly and strongly activated in maize seedlings by mannitol and salt. However, 30 min after ABA stimulation, this activity became barely detectable (Figure 5A) suggesting that ZmSNAC1 is phosphorylated by kinases transiently activated by hyperosmotic stress signals.

We then demonstrated that ZmSNAC1 can be directly phosphorylated by ZmOST1 *in vitro* (Figure 5B). To identify the ZmSNAC1 region required for ZmOST1 phosphorylation, we made three deletion constructs: two dividing the NAC-binding DNA domain that lacked the C-terminal regulatory region (construct A:1–100 aa and construct B: 101–170 aa) and one lacking the N-terminal NAC-binding DNA domain (construct C: 171–312 aa; Figure 5B). As shown in Figure 5B, only domain C was highly phosphorylated by ZmOST1 *in vitro* suggesting that it is the regulatory region which is phosphorylated by ZmOST1. These *in vitro* results are in accordance with our *in silico* prediction in which the domain C of ZmSNAC1 contains 12 potential phosphorylated peptides while ZmSNAC1-A and ZmSNAC1-B have two and one peptides, respectively (Table S1).

To ascertain whether ZmSNAC1 phosphorylation by ZmOST1 occurs and if other kinases could also phosphorylate this transcription factor, we used the heterologous *Arabidopsis* system described for the detection of the specific OST1/ZmOST1 activity (Figure 1B) on the recombinant ZmSNAC1 protein by modified in gel kinase assays. In these gels the MBP kinase substrate was replaced by SNAC1 overexpressed in *E. coli*. Total protein extracts from wild-type *Arabidopsis*, *ost1-2* mutant and transgenic *ZmOST1*/*ost1-2* seedlings treated or not with ABA were loaded and a phosphorylation assay was performed in gel. As shown in (Figure 5C), the *Arabidopsis* OST1 (42 kDa activity band) from ABA-treated wild-type plant extracts was capable of phosphorylating ZmSNAC1. Note that the same phosphorylation activity was clearly missing in extracts from the *ost1-2* allele [6] and it was recovered in extracts of *ZmOST1/ost1-2* complemented line (Figure 5C). To validate these results we immunoprecipitated the kinases from the same extracts using an antibody directed against the ZmOST1 ABA-box which recognizes OST1 and ZmOST1 proteins. As can be seen by the western blot (bottom gel), we recovered two proteins by immunoprecipitation using this antibody, the most abundant being OST1/ZmOST1. OST1 was recovered in similar amounts on the wild-type and *ost1-2* backgrounds and ZmOST1 in slightly increased quantities in the *ZmOST1/ost1-2* line used as a result of it being overexpressed under a *35S* promoter. Analyzing the in gel kinase assay presented in the OST1 immunoprecipitation followed by in gel kinase assay toward recombinant ZmSNAC1 (middle gel) we detected a band that was activated by ABA on the wild-type and *ZmOST1/ost1-2* line which was absent in the *ost1-2* mutant. This activity can only be identified as OST1/ZmOST1 [6] and indeed confirms that ZmOST1 can phosphorylate ZmSNAC1 by an ABA-dependent mechanism, possibly by enhancing the transcriptional activity or stability of ZmSNAC1 in response to ABA or osmotic stresses.

The above assays not only confirmed the phosphorylation of ZmSNAC1 but also show that several kinases of 60-, 45-kDa can also phosphorylate this transcription factor (Figure 5C). The 60-kDa kinase band probably represents the endogenous AKIN10, another stress-activated kinase of the SnRK1 family [52]. We have confirmed this by using ZmSNAC1 as substrate, and performing phosphorylation assays with total protein extracts from wild-type, *35S::AKIN10-HA* and *AKIN10 RNAi* seedlings (Figure S4) [53]. In contrast to the ABA-dependent phosphorylation of ZmSNAC1 by OST1/ZmOST1, AKIN10 phosphorylation occurs in the absence of ABA. Thus, the transcriptional activity or protein stability of ZmSNAC1 might be co-modulated by both ABA-dependent and ABA-independent signalling pathways.

### ZmOST1 alters the localization and stability of ZmSNAC1 under ABA treatment

**Figure 6:**
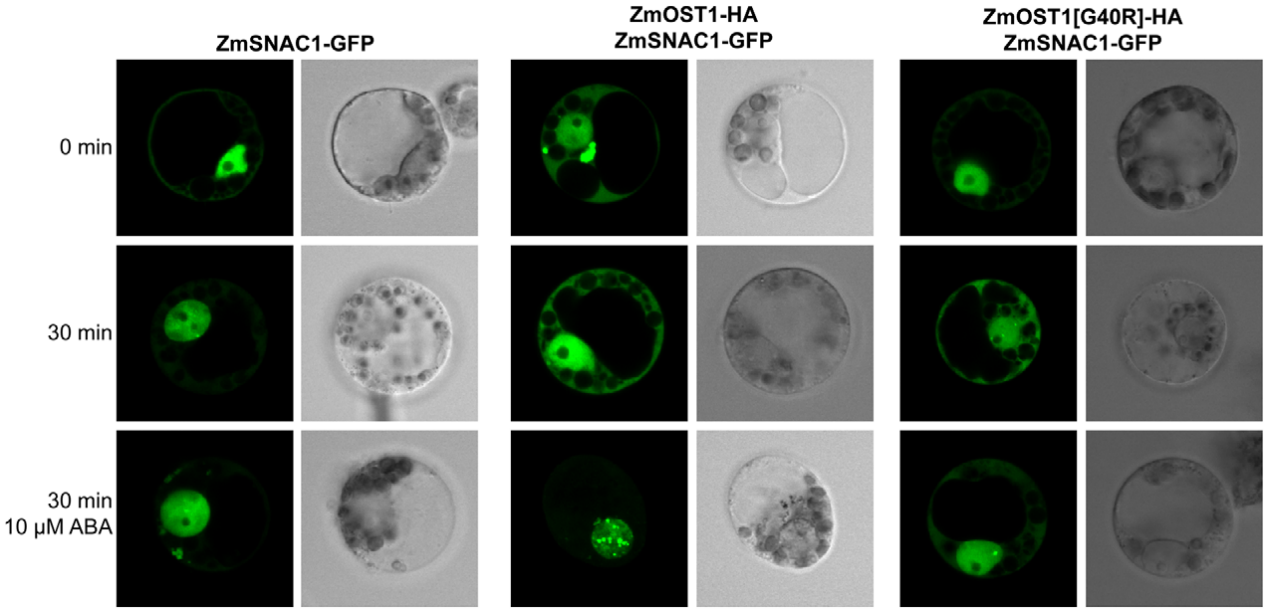
ZmSNAC1 localizes in nuclear speckles after ABA treatment when co-expressed with ZmOST1. ZmSNAC1-GPF localization is presented in the left, co-expressed with ZmOST1-HA in the middle and co-expressed with ZmOST1 [G40R]-HA in the right. Upper panel shows the localization of ZmSNAC1-GFP at the beginning of the experiment, centre panel the same localization after 30 minutes and the bottom panel represents ZmSNAC1-GFP localization after 30 minutes ABA treatment (10 µM).

Since ZmSNAC1 is phosphorylated by ZmOST1 after being activated by ABA, we were interested in determining the effects of this phosphorylation on ZmSNAC1, in particularly during ABA dependent signalling. We transiently co-expressed ZmSNAC1 fused to GFP in maize protoplasts together with ZmOST1 and ZmOST1 [G40R] fused to a HA-epitope and checked for fluorescence under a confocal microscope. Using this approach we were able to detect a change of localization of ZmSNAC1-GFP under ABA treatment when co-expressed with ZmOST1-HA to nuclear speckles that was absent when SNAC1-GFP was over-expressed alone (Figure 6). This formation of nuclear speckles is concomitant with a decrease of overall fluorescence that could have implications on protein stability. Furthermore, when we co-expressed ZmSNAC1 with the inactive ZmOST1 [G40R]-HA this speckled localization did not occur, giving strong indication that what we observed with the wild-type kinase was caused by ZmOST1 activity.

**Figure 7:**
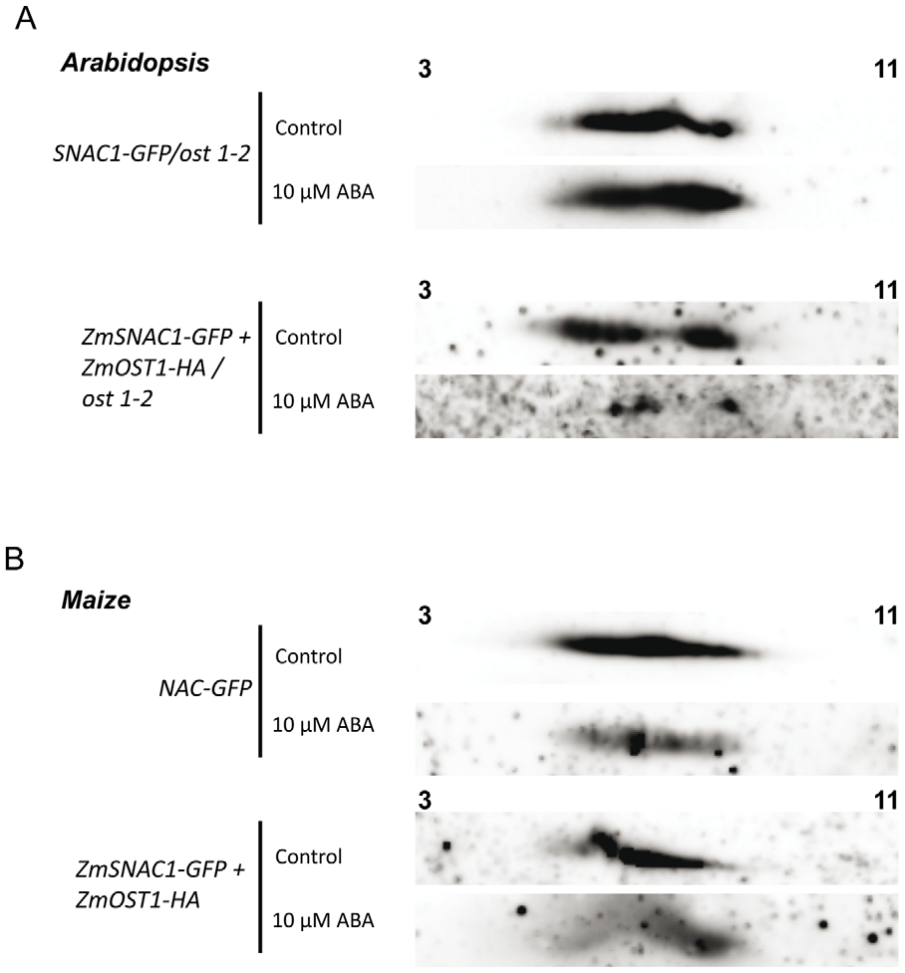
ZmOST1 alters ZmSNAC1 protein stability under ABA treatment. ZmSNAC1 phosphorylation and protein stability was analyzed by bi-dimensional gel electrophoresis followed by western blot. (**A**) *Arabidopsis ost1-2* mutant and (**B**) Maize B73 protoplasts transfected with ZmSNAC1-GFP alone or with ZmOST1-HA. Upper western blot corresponds to control situations the lower corresponds to 30 min 10 µM ABA treatment.

In order to better determine the *in vivo* phosphorylation and protein stability of SNAC1 we performed Bi-dimensional SDS-PAGE experiments comparing ZmSNAC1 protein fused to GFP using the *Arabidopsis* and maize protoplast systems. Even though in this experiment we were not able to clearly detect any protein shift that is consistent with a phosphorylation, the quantity and abundance of ZmSNAC1 spots is clearly affected by ABA treatment when ZmOST1 is present (Figure 7). While in the *ost1-2* protoplasts ZmSNAC1 quantity is unaffected by ABA, when protoplasts are co-transformed with ZmOST1, a clear reduction of the most acidic ZmSNAC1 spots is clear (Figure 7A). When repeating the experiment in maize protoplasts we were able to observe the same degradation of ZmSNAC1 under ABA treatment (Figure 7B). These results seem to indicate that ZmOST1 activity has an effect on ZmSNAC1 stability.

## Discussion

In recent years, significant progress has been made toward understanding the molecular basis for ABA signal transduction in *Arabidopsis* [25]. This has confirmed the SnRK2-related kinases, with OST1 being the founding member in *Arabidopsis*, as key elements in these responses [54]–[56].

The high homology of ZmOST1 with its *Arabidopsis* and rice counterparts (Figure S1) brought us to analyze the cellular function of ZmOST1 in stomatal closure. ZmOST1 complements the *Arabidopsis ost1-2* mutant. This allele carries a missense G33R mutation that renders the kinase catalytically dead, which translates into a strong phenotype of stomatal deregulation in response to both ABA and water stress signals [6]. Phenotypic complementation of this allele revealed that ZmOST1 promotes stomatal closure in response to water stress working as a positive regulator in the drought signalling cascade. In the *Arabidopsis* model for ABA signalling, the PYR/PYL/RCAR ligand inactivates the PP2C phosphatases in the presence of ABA, resulting in the rapid activation of SnRK2 kinases to regulate a large part of stress-responsive gene expression by reversible phosphorylation [57]–[59]. This stomatal closure response we report might be mediated by direct phosphorylation of ion transporters or of different transcription factors such as the nuclear AREBs/ABFs and a subfamily of the NAC type transcription factors such as NTL6 which has been described to be functionally associated with SnRK2.8 in drought stress response [22], [23], [60].

Although ABFs, the predicted major targets of OST1 regulation [22], are also expressed in the guard cell and regulate an estimated 80% of the global ABA transcriptome, the *abf2 abf3 abf4* triple mutant is normal in transpiration [22], [23], [25], [28], [61], [62] suggesting that other intermediates in the OST1-mediated transcriptional cascades in guard cell are still unknown. Among them, we identified ZmSNAC1. Unlike ABF which seem more involved in germination and seedling growth, SNAC proteins have a more prominent role in stomatal regulation [30]. Our results reveal that ZmSNAC1 becomes phosphorylated in response to osmotic stress and salt treatments in maize, suggesting that this posttranslational modification is needed to regulate its activation under stress [39]. One upstream kinase could well be ZmOST1.

ZmOST1/ZmSNAC1 interact in the nucleus (Figure 4), similar to what is described for OST1/ABF interaction in *Arabidopsis* [22] but different from SnRK2.8/NTL6 cytosolic interaction [60]. Whereas SnRK2.8 activity on NTL6 targets the transcription factor for nuclear import [60], it is possible that ZmOST1 phosphorylates SNAC1 to enhance its transcriptional activity through the regulation of protein stability, in a mechanism that could be opposite to what is described for the ABFs [22]. In the case of ABF3, phosphorylation by OST1 increases protein stability in what seems to be a two-step process that first accumulates and then activates the transcription factor to induce ABA regulated genes [22]. For ZmSNAC1, OST1 activity seems to be responsible for protein degradation. In this case, OST1 could rapidly and transiently regulate gene expression by first activating SNAC1 and then triggering the activated protein for degradation. Additional studies will be required to elucidate the function of ZmSNAC1 in abiotic stress responses.

Moreover, we showed that, in addition to ZmOST1 and other SnRK2s, an SnRK1-like activity could constitutively phosphorylate ZmSNAC1. SnRK1 protein kinases have also been shown to phosphorylate ABFs and both SnRK1 and SnRK2 kinases share targets of phosphorylation [20], [63], [64]. For these reasons, we cannot rule out that SnRK1 may also regulate SNAC1 activity together or alternately to ZmOST1.

The current model of ABA signalling mechanism reconstructed *in vitro* suggests that, in the absence of the hormone, the clade A protein phosphatases 2C inhibit OST1 activity by binding to its kinase catalytic site as well as to the ABA box, an acidic motif of ∼25 amino acids at the C-terminus of the kinase [49]. ABA triggers the pathway by binding to the PYL receptor, and the changes in the PYL conformation then allows it to insert its “proline gate” domain into the catalytic site of the phosphatase [50]. This dislodges the kinase but without dissociating the two proteins completely, as the kinase remains tethered by its ABA box to the phosphatase [50]. The attached protein kinase-phosphatase pair is thought to provide a rapidly reversible phospho-relay in regulating the on/off state of the ABA signalling pathway. *In vivo*, however, the association of the PP2C and SnRK2 (particularly OST1) can also be labile, because the complex can only be recovered after treatment of the total soluble protein extract from *Arabidopsis* by chemical cross-linkers [65] or transient over-expression of the two proteins in tobacco [66], [67]. Our current results could explain this labile interaction between the PP2C and OST1 during signalling. If the ABA box [49] is also the contact site for kinase substrates, complete dissociation of PP2C-SnRK would be possible from SNAC1 competition. This may be one mechanism by which the ABA signalling cascade can be sustained for long-term transcriptional reprogramming in guard cells, as opposed to rapid responses from ion transport.

ZmSNAC1 action could then be regulated by controlled degradation under ABA treatment by ZmOST1 mediated phosphorylation. In fact, enhancement of proteasome degradation of multiple factors by signal-induced phosphorylation has been already demonstrated [68], [69] and the proteolysis via the *26S* proteasome of critical regulators of other plant hormones has been established in the case of gibberellins [70], auxin [71], and jasmonate [72] which made some authors speculate that one or more proteins of the ABA receptor-signal complex and its downstream targets might be regulated by proteolysis [73]. After ZmSNAC1 degradation, ZmOST1 would be free to return to its repressed or on/off association with PP2C phosphatases.

Recent data showed that ZmSNAC1 and the rice homologs, *OsNAC5* and *OsNAC6*, highly induced by abiotic stresses are important for stress tolerance acquisition [38], [39], [74], [75]. Furthermore, over-expression of the homolog, *OsSNAC1*, enhances drought and salt tolerance in transgenic rice [35]. Importantly, over-expressing of SNAC1 did not engender phenotypic changes or yield penalty [35], which are important considerations for agronomic applications. The identity between rice and maize SNAC1 proteins suggests an evolutionarily conserved functional role in the transcriptional control of stomatal response although further experiments are necessary to demonstrate whether enhanced expression of *ZmSNAC1* would also augment drought tolerance in maize without undesirable phenotypic alterations.

## Materials and Methods

### Ethics statement

N/A – No animal trials were conducted in this paper. Antibody production was outsourced as a service to CID-CSIC.

### Plant material

*Arabidopsis* plants were maintained in controlled growth chambers (24±2°C, 16 h∶8 h light:dark photoperiod). Seeds were germinated in medium containing 1× MS basal salt mixture supplemented with 0.05% MES and 1% sucrose. Seeds were incubated at 4°C for 3 days to break dormancy prior to germination. ABA treatment was carried out at 100 µM. The *akin10 Arabidopsis* overexpression and RNAi lines were kindly provided by Dr. Elena Baena [53].

### Full-length ZmOST1 cDNAs isolation

A phagemid cDNA library constructed from poly(A)^+^ RNA maize water-stressed leaves was screened by filter hybridization under high-stringency conditions, using a [α-^32^P]-labelled partial probe corresponding to the *ZmOST1* gene (ACG36261).

### ZmOST1 polyclonal antiserum production

The recombinant ZmOST1 and ABA-box (325–366 aa) proteins fused to a six histidine tag (6×His) in their amino terminal region were expressed and purified according to the pET manual (Novagen). Purified ZmOST1 fusion protein was used to produce polyclonal antisera by an external service (CID-CSIC). Detection of ZmOST1 fused protein was estimated with a dot blot analysis on nitrocellulose membranes.

### Immunoprecipitation experiments

*Arabidopsis* proteins were extracted in 50 mM HEPES pH 7.5, 5 mM EDTA, 5 mM EGTA, 10 mM DTT, 10 mM Na_3_VO_4_, 10 mM NaF, 50 mM β-glycerolphosphate, 1 mM PMSF, 10 μM Leupeptin, 2 μg/ml Aprotinin and 10 μg/ml Pepstatin and cleared twice by centrifugation at 13000 rpm at 4°C for 15 min. *Arabidopsis* (500 μg) extracts were incubated with a 1∶100 dilution of the ABA-box (325–366 aa) antiserum in 300 µl of IP buffer (20 mM Tris-HCl pH 7.5, 1 mM EDTA, 1 mM EGTA, 2 mM DTT, 2 mM Na_3_VO_4_, 2 mM NaF, 10 mM β-glycerolphosphate, 150 mM NaCl, 0.5% [v/v] Triton X-100, 0.5% [v/v] Nonidet NP40, 1 mM PMSF, 10 μM Leupeptin, 2 μg/ml Aprotinin and 10 μg/ml Pepstatin). After 3 h in a rotary shaker, 40 μl of protein A-Sepharose CL-4B 50% slurry (GE Healthcare) was added and incubated for another hour. The slurry was washed 3×15 min with IP buffer and the supernatant was removed prior to the in gel kinase assay.

### In-gel kinase assay

Protein samples were separated on 10% SDS-PAGE gels embedded with 0.25 mg/ml MBP as substrate. In gel kinase assay was performed according to [10]. Gels were washed with 25 mM Tris-HCl pH 7.5, 0.5 mM DTT, 0.1 mM Na_3_VO_4_, 5 mM NaF, 0.5 mg/ml BSA and 0.1% Triton X-100 for 3×30 min at room temperature, and proteins were renatured with 25 mM Tris-HCl pH 7.5, 1 mM DTT, 0.1 mM Na_3_VO_4_ and 5 mM NaF for 2×30 min and 16 h at 4°C. Kinase activity was assayed in 25 mM Tris-HCl pH 7.5, 1 mM DTT, 2 mM EGTA, 0.1 mM Na_3_VO_4_, 12 mM MgCl_2_, 250 nM cold ATP and 100 μCi [γ-^33^P]-ATP at room temperature for 1 h. Finally, gels were washed extensively with 5% [w/v] TCA, 1% Na_2_PPi solution at least five times and dried. Radioactivity was detected using a Storm 820 imager (GE Healthcare). For ZmSNAC1 kinase assay, 0.5 mg/ml of HIS-tagged protein were embedded in the SDS-PAGE gels and 40 µg of maize and *Arabidopsis* extracts were loaded for each lane.

### Transgenic *Arabidopsis* plants generation and analyses

Full-length *ZmOST1* cDNA were amplified by PCR using oligonucleotides 5′-CCGAATTCATGGCAGGGCCGGCGCCG-3′ and 5′-GGCTCGAGTCACATTGCGTATACAATCTCAC-3′. The PCR product was cloned into the pGEM-T Easy vector (Promega), digested BamHI/XhoI and subcloned into the pBinAr vector under the CaMV 35S promoter. This construct was used to transform by floral dip infiltration using *Agrobacterium tumefaciens* (C_58_C_1_) wild type (Ler) and *Arabidopsis ost1-2* mutant plants [6]. Transgenic seedlings selection was performed in 0.5× MS solid medium supplemented with 1% sucrose, 0.5 g/l MES and 50 mg/l kanamycin at 21°C under a long-day photoperiod. Five *35S::ZmOST1/ost1-2* homozygous transgenic lines per construct and genetic background were analysed for transgene expression by reverse transcription PCR (RT-PCR) with similar *ZmOST1* expression results opting for lines 211 and 287 for further analyses. The inserted *ZmOST1* transgene was amplified with oligonucleotides 5′-GTAAGAACGTGCGATTCAGTG-3′ and 5′-TATCATGCGATCATAGGCGTC-3′. Water-loss experiments were only performed with *ZmOST1* overexpression in the *ost1-2* allele due to the lack of expression obtained in transgenic lines overexpressing *ZmOST1* in the wild-type background (data not shown).

Water-loss measurements were performed with 2 weeks-old *Arabidopsis* plants grown routinely on soil. For each genotype, three rosettes were detached and weighted during 3 h in intervals of 10 min. Water loss was calculated as percentage of weight at the indicated times in relation to the initial fresh weight. Thermal images of *Arabidopsis* leaves were taken using a Thermacam PM250 infrared camera (Inframetrics) 5 min after they were detached from 1 week old plantlets grown in a phytotron at 21°C under a long-day photoperiod and 70% relative humidity [76].

### Yeast two-hybrid screening and *in vitro* pull-down experiments

Full-length *ZmOST1* cDNA was amplified by PCR and subcloned into the vector pGBT7 (Clontech). A maize cDNA library from 5-day-old leaves water-stressed for 3 h was constructed using the activation domain expression vector pAD-GAL4 [77] (Stratagene). pGBT7-ZmOST1 construct was transformed directly into *Saccharomyces cerevisiae* AH109 strain. Yeast expressing ZmOST1 protein was retransformed with the pADGAL4-cDNA maize library, as previously described [77], [78]. For yeast interaction experiments ZmSNAC1 was cloned in the vector pGAD424. β-galactosidase liquid assays were performed as described by [79]. For pull-down experiments, *ZmOST1* cDNA was cloned into pGEX4T (GE Healthcare) and *ZmSNAC1* cDNA into the pET28a (Promega) expression vectors. Binding assays were performed with equal amounts of GST proteins and 20–30 μl of^35^S-labeled ZmSNAC1 protein synthesized using the TNT-coupled rabbit reticulocyte lysate system (Promega), in 180 μl of binding buffer (20 mM HEPES-KOH (pH 7.9), 50 mM KCl, 2.5 mM MgCl_2_, 10% glycerol, 1 mM DTT, 0.2% NP-40). Binding reactions were rolled overnight at 4°C, and washed four times with 1 ml of RIPA buffer [10 mM Tris-HCl (pH 7.5), 150 mM NaCl, 1 mM EDTA, and 0.2% NP-40]. The beads were boiled for 1 min in sample buffer and aliquots examined by electrophoresis as described by [80].

### GFP localization and BiFC by confocal microscopy

Full-length *ZmOST1* and *ZmSNAC1* cDNAs were cloned in the PC1302 vector (Clontech) and in the GATEWAY-compatible vector pENTRY3C (Invitrogen). A point mutated G40R ZmOST1 protein and different ZmOST1 protein domains corresponding to the catalytic-osmotic region (1–289 aa), the regulatory domain (290–366 aa), the osmotic SnRK2 box (290–325 aa) and the ABA box (325–366 aa) were also cloned in pENTRY3C vector. The six pENTRY3C plasmids were transferred to pYFN43 and pYFC43 BiFC GATEWAY-modified vectors described in http://www.ibmcp.upv.es/FerrandoLabVectors.php to produce 35S::YC-ZmOST1; 35S::YC-ZmOST1 [G40R]; 35S::YC-ZmOST1 [1–289]; 35S::YC-ZmOST1 [290–366]; 35S::YC-ZmOST1 [290–325]; 35S::YN-ZmOST1 [325–366] and 35S::YC-ZmSNAC1. *Nicotiana benthamiana* plants were transiently transfected with these constructs. For the co-infiltration, equal volumes of the three *Agrobacterium* cultures (the two truncated YFP constructs; and the strain expressing the HcPro protein) were mixed [81]. Confocal observations were performed 3 days after infiltration.

### *In vitro* phosphorylation

cDNA fragments encoding for ZmOST1, ZmSNAC1, and three ZmSNAC1 derivatives corresponding to A, B and C domains (1–100; 101–170; 171–312 aa, respectively) were cloned into the pET28a expression vector (Promega), expressed in *Escherichia coli* BL21 cells and purified as His-tag fusion proteins according to the manufacturers' instructions. Purified *E. coli*-expressed ZmOST1 (500 ng) were incubated at 30°C for 30 min with either 500 ng of purified ZmSNAC1 or of the truncated A, B and C domains in a final 15 μl volume of 1X kinase buffer (20 mM HEPES pH 7.5, 1 mM DTT, 10 mM MgCl_2_, 5 mM NaF, 125 mM β-glycerolphosphate), 25 μM cold ATP and 5 μCi [γ-^33^P]-ATP). Relative ^33^P incorporation was analyzed using the image analysis program ImageJ.

### Transient expression assays using maize and *Arabidopsis* leaf protoplasts

Transient expression of maize protoplasts was performed as previously described [82] with some modifications. Protoplasts from 11 to 13 days old etiolated maize seedlings were obtained from kernels of B73 plants. After chopping second or third leaves into small pieces, leaf stripes were digested in 3% cellulase onozuka R10, 0.6% macerozyme R10 (Yakult Honsha Co.), 0.6 M mannitol, 10 mM MES, pH 5.7, 5 mM CaCl2, and 0.1% (w/v) BSA for 15 min under vacuum followed by 150 min gentle shaking at 50 rpm in the dark at 28°C. After releasing the protoplasts at 90 rpm for 30 min, the protoplasts were filtered through a 35-mm nylon mesh and collected by centrifugation. The protoplasts were washed twice in 0.6 M mannitol, 5 mM MES, pH 5.7, and 10 mM KCl and counted with a hemocytometer. Electroporation was performed on 1–2×10^5^ protoplasts per transformation with 20 μg of plasmid DNA (100 V/cm, 200 μF) with a Biorad Gene Pulser II, high capacitance. After electroporation, protoplasts were incubated for 24 h in the dark at room temperature prior to analysis.

Transient gene expression on *Arabidopsis* mesophyll protoplast was performed according to the Sheen lab protocol [83] on well-expanded leaves from 3-week-old Arabidopsis plants grown on short day conditions (8 h light:16 h dark).

Treatment of maize and *Arabidopsis* protoplast was performed for 30 minutes by adding 10 µM ABA.

### Bi-dimensional gel electrophoresis

For two dimensional gel electrophoresis, transfected protoplasts were solubilised in 7 M urea, 2 M thiourea, 4% CHAPS, 4% Triton X-100, 18 mM Tris-HCl pH 8 in the presence of 53 u/ml DNase I, 4.9 u/ml RNaseA, 1 mM PMSF, 50 μM leupeptin, 1 μM pepstatin, 10 μM E-64 and 10 μg/ml aprotinin and cleared by centrifugation at 13000 rpm at 4°C for 5 min.

Total protein (60 μg) was diluted in rehydration solution (8 M Urea, 18 mM Tris-HCl, pH 8.0, 4% w/v CHAPS, 0.5% v/v IPG buffer (pH 3–11), 1.6% v/v DeStreak Reagent (GE Healthcare) and 0.002% w/v Bromophenol Blue) and loaded onto 7 cm IPG strips (NL pH 3–11) (GE Healthcare). Strips were rehydrated for 6 h at room temperature, followed by 6.5 h at 30 V. IEF was performed at 500 V (1 h), 1000 V (1 h) and 8000 V (7 h) using the Ettan™ IPGphor™ Isoelectric Focusing System (GE Healthcare). Prior to second dimension, strips were equilibrated with 50 mM Tris-HCl (pH 8.8), 6 M urea, 30% v/v glycerol, 2% v/v SDS, a trace of Bromophenol Blue and 10 mg/mL DTT (15 min), followed by a second equilibration step (25 mg/mL iodoacetamide, 15 min). For the second dimension, proteins were separated on 12% SDS-PAGE gels. Western blot was performed as indicated previously using the Living Colors A. v. Monoclonal JL-8 GFP antibody (Clontech).

## Supporting Information

Figure S1 **ZmOST1 is the maize homolog of the** ***Arabidopsis*** **OST1 and the rice SAPK8.** ZmOST1 shares 83% identity with OST1 and 95% identity with SAPK8 indicating a potential conserved function on drought and osmotic stress response, namely at the level of stomata.
(TIF)

Figure S2 **ZmOST1 is constitutively expressed in maize.** (A) ZmOST1 expression of maize seeds collected at 14, 16, 20, 30, 40 and 60 days after pollination (dap) and seedlings at 1, 2 days after imbibition (dai). ZmOST1 expression is analyzed by Northern-blot experiments using RAB17 and ethidium bromide-stained ribosomal (18S) RNA genes as controls for ABA level and loading, respectively (B) ZmOST1 expression of maize seedlings in response to ABA, drought, NaCl and mannitol treatments.
(TIF)

Figure S3 **The G40R mutation of ZmOST1 renders the kinase inactive.** In vitro phosphorylation of MBP by ZmOST1 and ZmOST1 (G40R) reveals that the mutated kinase is unable to auto-phosphorylate or trans-phosphorylate MBP.
(TIF)

Figure S4 **Phosphorylation of ZmSNAC1 by AKIN10.** In gel kinase assay with proteins extracted from seedlings of wild-type (Ler), *35S::AKIN10-HA* (OX2) and AKIN10-RNAi (RNAi7) transgenic lines (Baena-González et al., 2007) using ZmSNAC1 as substrate. Sizes of activity bands are shown on the left. The 60 Kd band probably represents the kinase activities of the endogenous AKIN10 and the closely related AKIN11, kinases with a similar MW of approximately 60 Kd (Zhang et al., 2009). A strong band of activity of about 66 Kd is obtained in extracts from *35S::AKIN10-HA* seedlings suggesting that AKIN10 is likely to phosphorylate ZmSNAC1 protein *in vivo* together with other kinases (45 Kd activity band; asterisk).
(TIF)

Table S1 **Predicted ZmOST1 phosphorylation loci on the ZmSNAC1 sequence using the web based bioinformatics tool Predikin.** Two putative peptides are found on SNAC1-A, one on SNAC1-B and 12 on SNAC1-C.
(TIF)
