# Supplementary figures and images for: The Maize OST1 Kinase Homolog Phosphorylates and Regulates the Maize SNAC1-Type Transcription Factor
Source: PLoS One. 2013 Feb 28;8(2):e58105. doi: 10.1371/journal.pone.0058105 (PMC3585266; doi:10.1371/journal.pone.0058105)

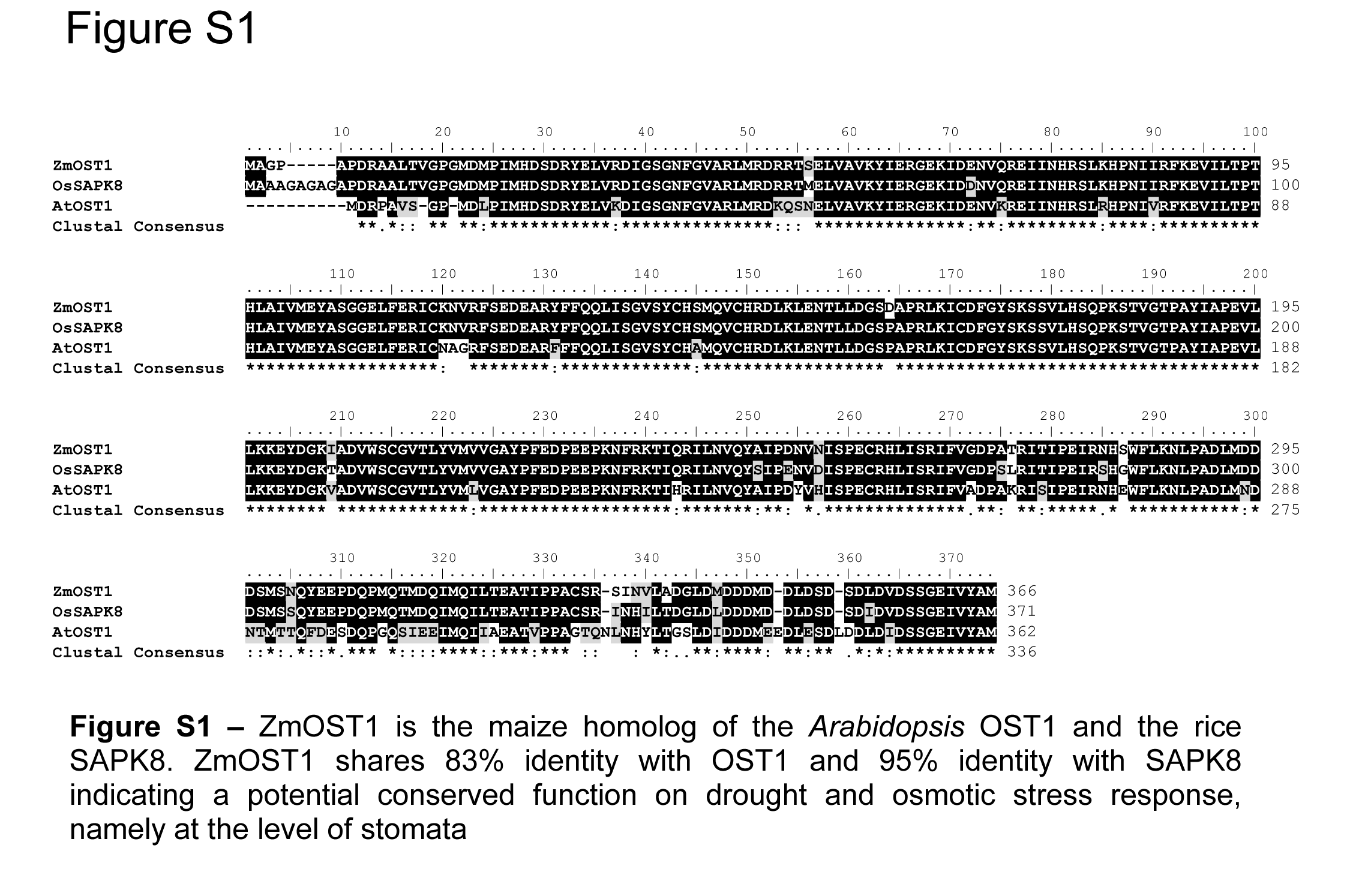

Supplement: Figure S1 — ZmOST1 is the maize homolog of the Arabidopsis OST1 and the rice SAPK8. ZmOST1 shares 83% identity with OST1 and 95% identity with SAPK8 indicating a potential conserved function on drought and osmotic stress response, namely at the level of stomata. (TIF) [file pone.0058105.s001.tif]

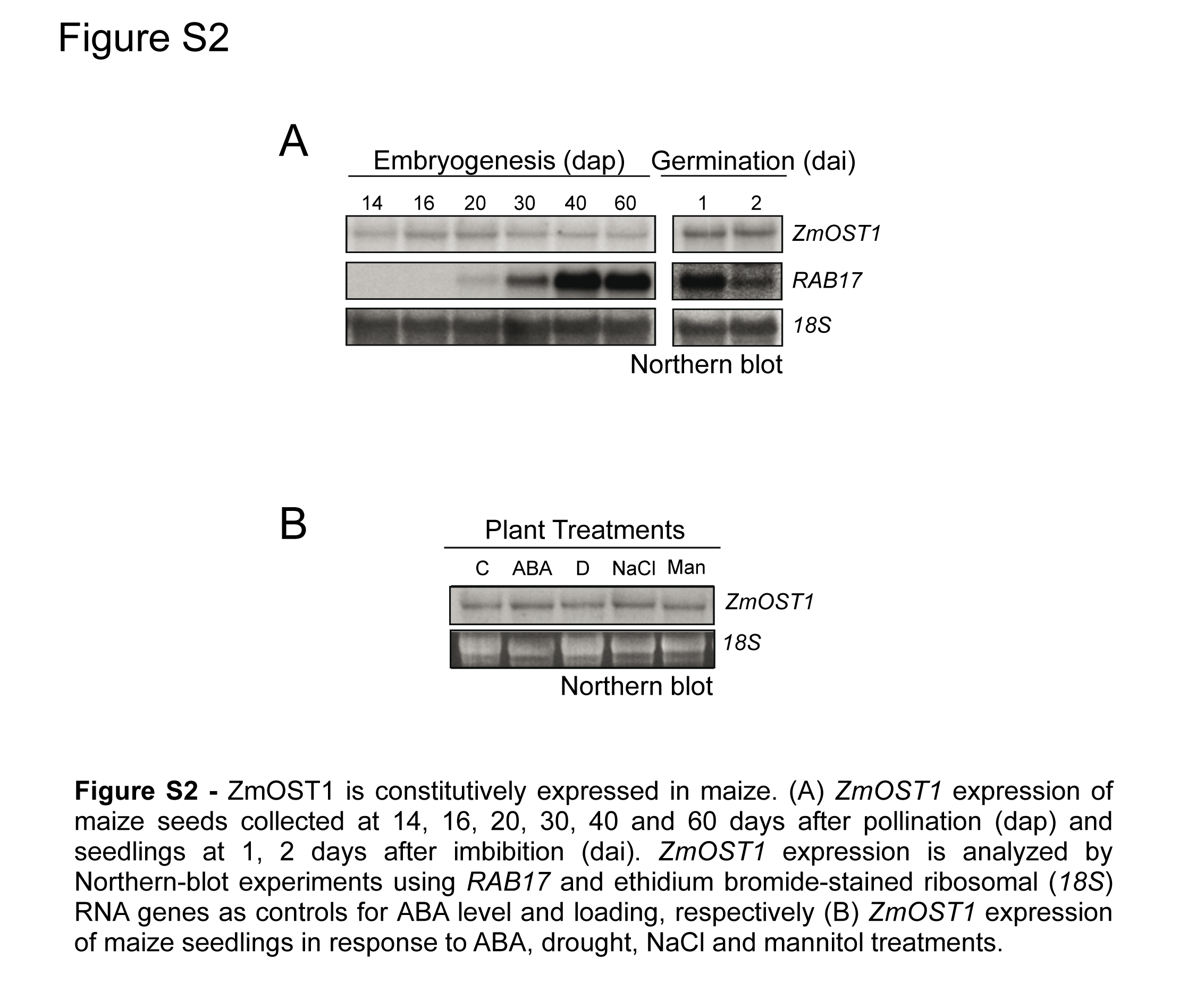

Supplement: Figure S2 — ZmOST1 is constitutively expressed in maize. (A) ZmOST1 expression of maize seeds collected at 14, 16, 20, 30, 40 and 60 days after pollination (dap) and seedlings at 1, 2 days after imbibition (dai). ZmOST1 expression is analyzed by Northern-blot experiments using RAB17 and ethidium bromide-stained ribosomal (18S) RNA genes as controls for ABA level and loading, respectively (B) ZmOST1 expression of maize seedlings in response to ABA, drought, NaCl and mannitol treatments. (TIF) [file pone.0058105.s002.tif]

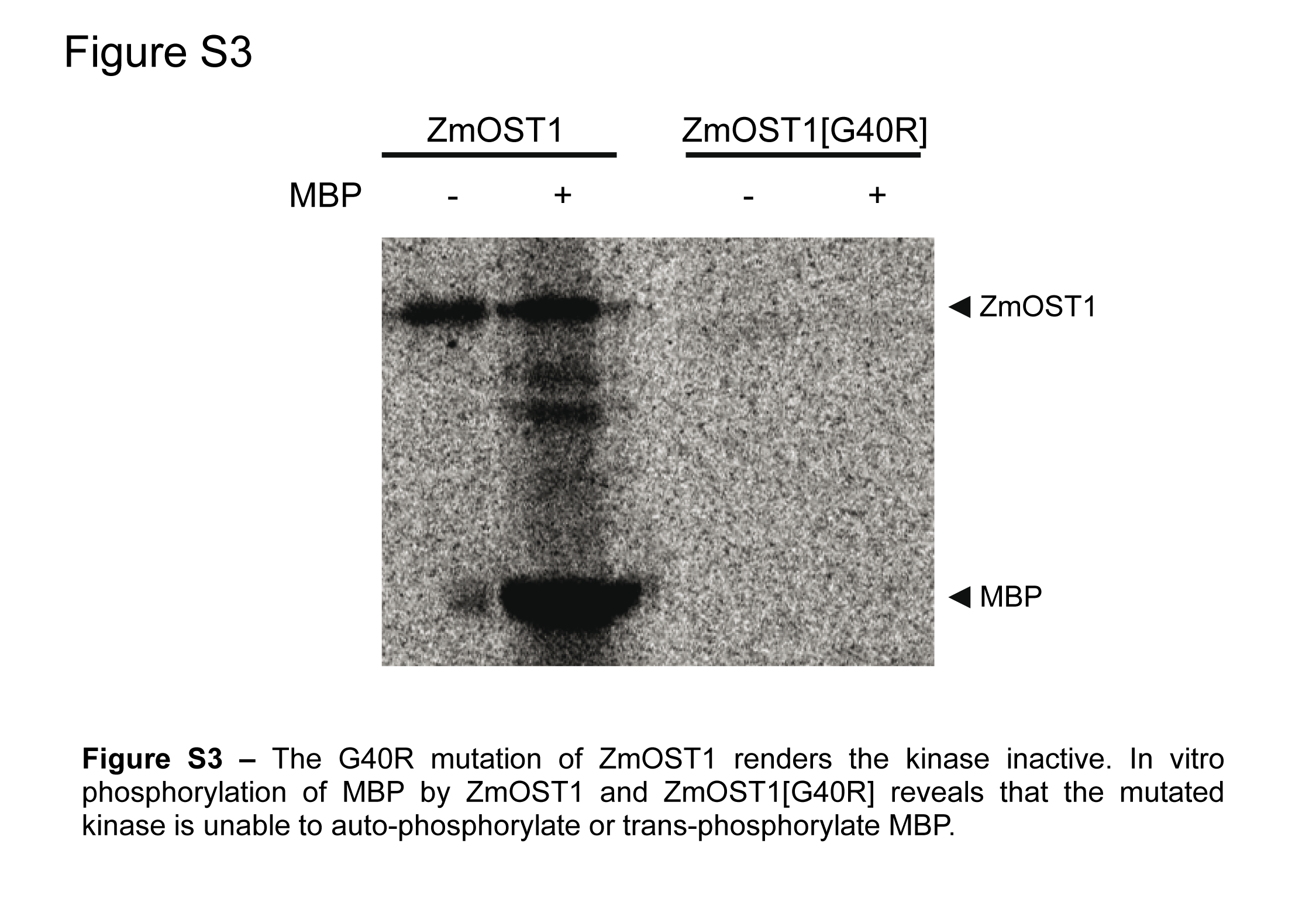

Supplement: Figure S3 — The G40R mutation of ZmOST1 renders the kinase inactive. In vitro phosphorylation of MBP by ZmOST1 and ZmOST1 (G40R) reveals that the mutated kinase is unable to auto-phosphorylate or trans-phosphorylate MBP. (TIF) [file pone.0058105.s003.tif]

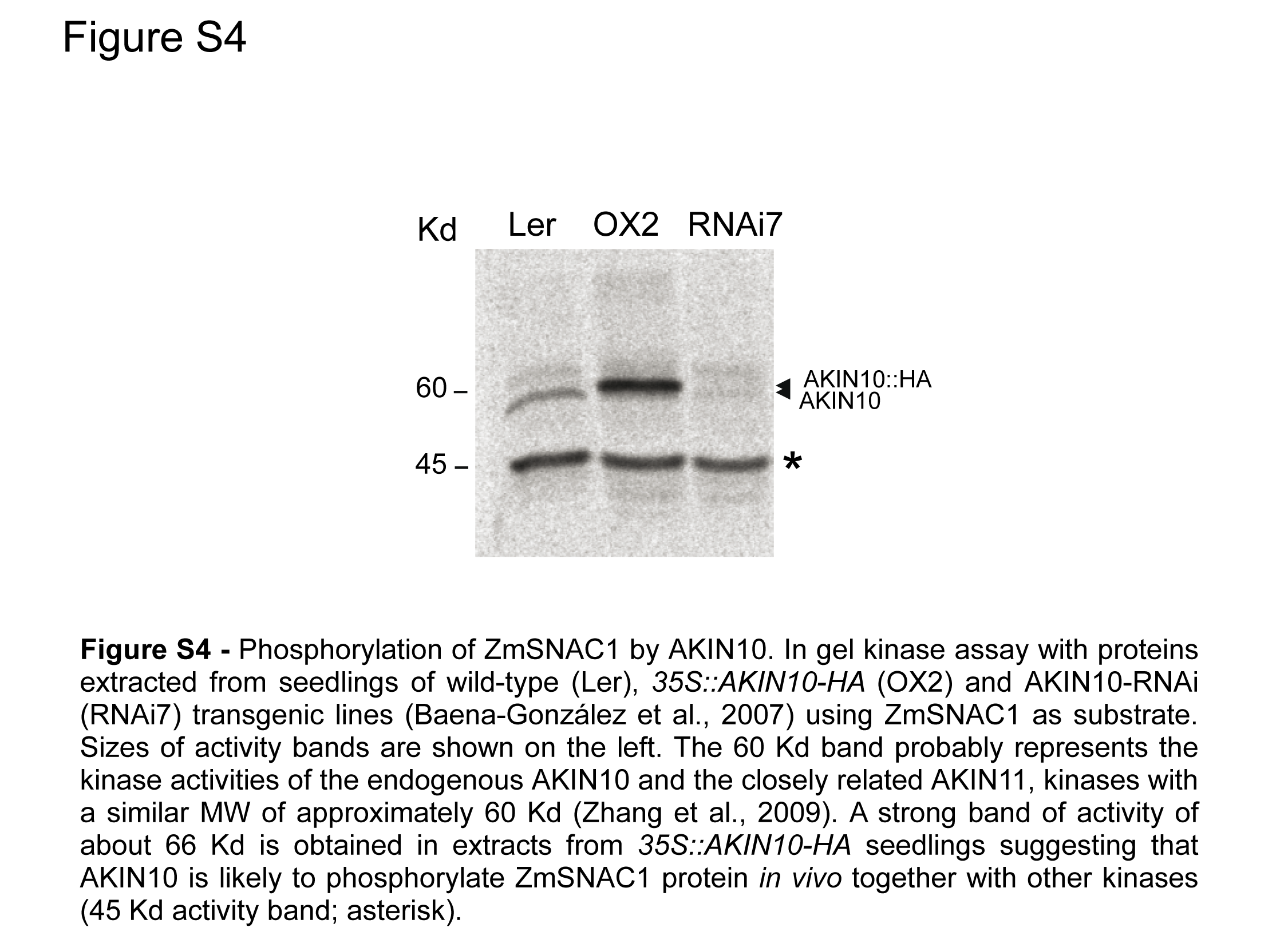

Supplement: Figure S4 — Phosphorylation of ZmSNAC1 by AKIN10. In gel kinase assay with proteins extracted from seedlings of wild-type (Ler), 35S::AKIN10-HA (OX2) and AKIN10-RNAi (RNAi7) transgenic lines (Baena-González et al., 2007) using ZmSNAC1 as substrate. Sizes of activity bands are shown on the left. The 60 Kd band probably represents the kinase activities of the endogenous AKIN10 and the closely related AKIN11, kinases with a similar MW of approximately 60 Kd (Zhang et al., 2009). A strong band of activity of about 66 Kd is obtained in extracts from 35S::AKIN10-HA seedlings suggesting that AKIN10 is likely to phosphorylate ZmSNAC1 protein in vivo together with other kinases (45 Kd activity band; asterisk). (TIF) [file pone.0058105.s004.tif]

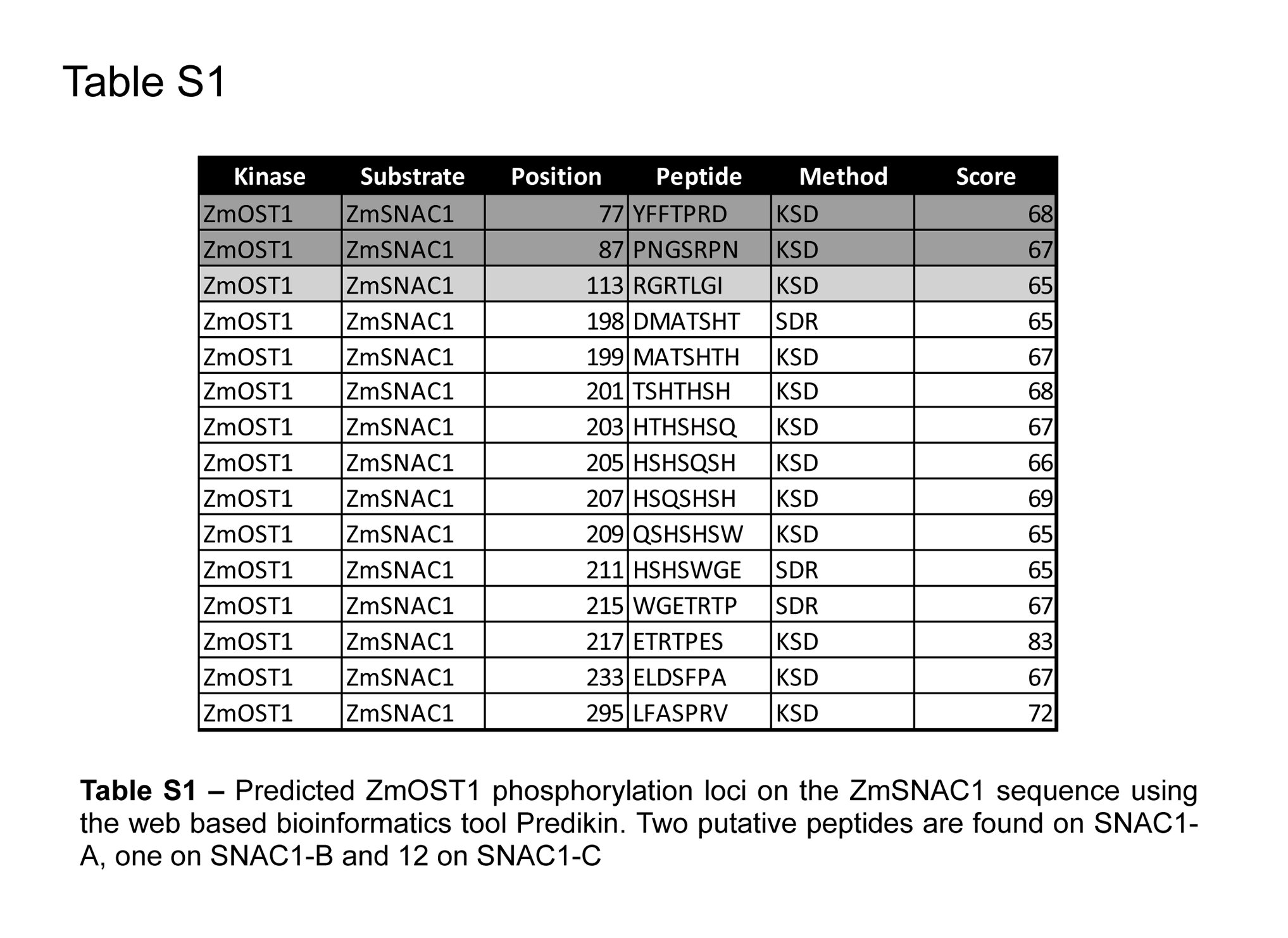

Supplement: Table S1 — Predicted ZmOST1 phosphorylation loci on the ZmSNAC1 sequence using the web based bioinformatics tool Predikin. Two putative peptides are found on SNAC1-A, one on SNAC1-B and 12 on SNAC1-C. (TIF) [file pone.0058105.s005.tif]
